# Usefulness of FDG PET/CT derived parameters in prediction of histopathological finding during the surgery in patients with pancreatic adenocarcinoma

**DOI:** 10.1371/journal.pone.0210178

**Published:** 2019-01-10

**Authors:** Altay Myssayev, Ayan Myssayev, Reiko Ideguchi, Susumu Eguchi, Tomohiko Adachi, Yorihisa Sumida, Shuichi Tobinaga, Masataka Uetani, Takashi Kudo

**Affiliations:** 1 Department of Radioisotope Medicine, Nagasaki University Graduate School of Biomedical Sciences, Nagasaki, Japan; 2 Public Health Department, Semey State Medical University, Semey City, Republic of Kazakhstan; 3 Department of Radioisotope Medicine, Atomic Bomb Disease Institute, Nagasaki University, Nagasaki, Japan; 4 Department of Surgery, Nagasaki University Graduate School of Biomedical Sciences, Nagasaki, Japan; 5 Department of Surgical Oncology, Nagasaki University Graduate School of Biomedical Sciences, Nagasaki, Japan; 6 Department of Surgery, Sasebo City General Hospital, Nagasaki, Japan; 7 Department of Radiological Sciences, Nagasaki University Graduate School of Biomedical Sciences, Nagasaki, Japan; Biomedical Research Foundation, UNITED STATES

## Abstract

**Purpose:**

Pancreatic cancer is the 4th most common cause of cancer death in Japan and exhibits a 5-year overall survival rate of approximately 7%. The accurate diagnosis of pancreatic cancer is important for determining the optimal management strategy. Fludeoxyglucose-positron emission tomography (FDG PET) integrated with computed tomography (^18^F-FDG PET/CT) has emerged as a powerful imaging tool for detecting and evaluating various cancers, and it is used for staging, detecting local recurrence and distant metastasis, measuring therapeutic effects, and predicting prognosis in pancreatic cancer patients.

Lately, FDG PET/CT-derived parameters, such as standardized uptake values (SUV), the metabolic tumor volume (MTV), and total lesion glycolysis (TLG), have been suggested as prognostic factors for various types of cancer, including pancreatic cancer. However, there is no consensus regarding the best parameters for evaluating patient prognosis, operability, etc. The purpose of this study was to examine the differences between operable and non-operable pancreatic cancer using FDG PET/CT-derived parameters, and to investigate whether volumetric parameters (TLG and the MTV) are superior to SUV-based parameters for predicting infiltration status/determining operability.

**Materials and methods:**

We conducted a retrospective study of the cases of 48 patients with clinically proven pancreatic adenocarcinoma, who underwent FDG PET/CT imaging before treatment. In the operable group, the surgical specimens were subjected to histopathological examinations, and the cases were separated into those exhibiting less and greater infiltration. SUVmax, SUVpeak, the tumor background ratio (TBR), TLG, and the MTV were compared between these groups as well as between the operable and non-operable groups.

**Results:**

Venous infiltration showed significant associations with several metabolic parameters (SUVmax, SUVpeak, and the TBR). However, it did not display any significant associations with volumetric parameters, such as TLG or the MTV. None of the FDG PET/CT-derived parameters exhibited significant associations with lymphatic or neural infiltration. Significant differences in volumetric parameters, such as the MTV and TLG, were detected between the operable and non-operable subgroups.

**Conclusions:**

Metabolic ^18^F-FDG PET/CT-derived parameters, such as SUVmax, SUVpeak, and the TBR, are useful for predicting venous infiltration status in patients with operable pancreatic adenocarcinoma.

## Introduction

Pancreatic cancer causes 265,000 cancer deaths annually in developed countries [1], and it ranks as the 4^th^ leading cause of cancer deaths in Japan. In 2015, 38,700 new cases of pancreatic cancer were diagnosed, and 32,800 patients died from this disease in Japan[2]. The poor prognosis of pancreatic cancer is caused by its highly aggressive nature and its tendency for invasive growth (the depth of invasion is a prognostic indicator) [3] and early dissemination to regional (e.g., the lymph nodes) and distant (e.g., the liver, peritoneum, or lungs) sites. The non-specific and late nature of its symptoms also make a poor prognosis more likely.

Surgical resection is the mainstay of curative treatment and offers the only chance of long-term survival in patients with small localized pancreatic tumors [4]. Unfortunately, only 10–20% of patients present with operable tumors [5], [6]. The remaining patients have inoperable locally advanced or metastatic pancreatic adenocarcinoma. This group of patients is usually treated with a combination of chemotherapy and radiotherapy. Accurate imaging of pancreatic cancer is essential for facilitating appropriate initial decisions regarding surgical management and might help to guide the appropriate use of other therapies, such as chemotherapy and radiotherapy [7]. Fluorine-18 fluorodeoxyglucose positron emission tomography (^18^F-FDG PET) has been demonstrated to be useful for determining the stage of the disease, detecting local recurrence and distant metastasis, assessing therapeutic effects, and predicting prognosis in pancreatic cancer patients [8–10].

FDG PET is a nuclear imaging technique, which is able to detect proliferating tumors based on their increased uptake and metabolism of glucose. Pancreatic tumor cells usually exhibit high glycolytic activity. As ^18^F-FDG is a radiolabeled analog of glucose, ^18^F-FDG PET reveals tumors by highlighting areas of increased glycolytic activity in vivo, which allows the 3D visualization of glucose metabolism. Nowadays, ^18^F-FDG PET/computed tomography (CT) is widely used to evaluate many different types of malignancy, including pancreatic cancer. FDG PET has higher sensitivity and specificity for staging pancreatic adenocarcinoma compared with CT and magnetic resonance imaging (MRI) [11], [12]. It has been established that MRI and contrast-enhanced CT are useful imaging techniques for the initial staging of pancreatic adenocarcinoma. ^18^F-FDG PET has the advantage of providing scans of the whole body in one session, which allows the initial staging to be performed and reveals any distant metastasis or nodal involvement [5], [6]^,^[13], [14]. Furthermore, ^18^F-FDG PET is able to detect tumors earlier than conventional imaging techniques and can be used to evaluate tumor aggressiveness and predict prognosis.

Some PET-related parameters are indicative of tumor activity. PET images are usually converted to standardized uptake values (SUV). SUV are calculated as the tissue activity concentration (Bq/ml) multiplied by the patient’s body weight (g) divided by the injected dose (Bq), and hence, they account for inter-individual differences in body mass and the injected tracer dose. Numerous parameters can be calculated based on SUV. The SUVmax of a tumor is defined as the maximum uptake value among all voxels. The SUVpeak of a tumor is defined as the average SUV for a 1-cm^3^ spherical volume around the SUVmax. It aims to minimize the fluctuations in measured activity caused by poor signal/noise ratios, which can affect the SUVmax. The mean SUV (SUVmean) of the liver (as a normal organ) is used to calculate the tumor background ratio (TBR), which is defined as the ratio of the lesion’s SUVmax to the SUVmean of the healthy liver (background). In addition, various volumetric parameters can also be obtained from FDG PET images. For example, the metabolic tumor volume (MTV; cm^3^) is defined as the volume of the tumor that exhibits FDG uptake. Unlike SUVmax, which represents a tumor’s maximum single-voxel FDG uptake value, the MTV quantifies the overall tumor burden. In addition, total lesion glycolysis (TLG; g) can be calculated using the following formula: the SUVmean of the tumor x the MTV. Recently, FDG PET/CT-derived parameters, such as SUV, the MTV, and TLG, have been suggested as prognostic factors for various tumors, including pancreatic cancer [15–17].

As mentioned above, there are many parameters, including both volumetric and metabolic parameters, that can be used to assess pancreatic cancer. However, there is no consensus regarding the best parameter for evaluating the prognosis or operability of pancreatic cancer. The purpose of this study was to examine the differences between operable and non-operable pancreatic cancer using FDG PET/CT-derived parameters, and to investigate whether volumetric parameters (TLG and the MTV) are superior to SUV-based parameters for assessing the infiltration status/operability of pancreatic cancer.

## Materials and methods

### Ethics

All procedures performed in studies involving human participants were in accordance with the ethical standards of the institutional and/or national research committee and with the 1964 Helsinki declaration and its later amendments or comparable ethical standards. The protocol was approved by the ethics committee of Nagasaki university hospital (Protocol Number: 16122621). The ethics committee of Nagasaki university hospital waived the need of informed consent from participants because this research was a retrospective and non-invasive, and the identifying information was not included in the collected data.

### Patients

We conducted a retrospective study of the cases of 48 patients with clinically proven pancreatic adenocarcinoma who underwent FDG PET imaging before treatment in the period between June 2010 and April 2016 at Nagasaki University Hospital. There were 27 males and 21 females, and their mean age was 68.2 years (range, 40–86 years). All of the patients who had biopsy-proven pancreatic adenocarcinoma and underwent FDG PET examinations at our hospital were included in the study. Patients who underwent surgical interventions, radiotherapy, or chemotherapy before the FDG PET examination were excluded. All patients with pancreatic adenocarcinoma were subjected to medical examinations and then were separated into operable and non-operable groups according to the clinical decisions of the attending doctors.

The Institutional Review Board waived the need of informed consent from participants because this research was a retrospective and non-invasive, and the identifying information was not included in the collected data.

### PET/CT protocol

^18^F-FDG PET was performed with a PET scanner (Siemens mCT, Germany). All patients were instructed to refrain from consuming food and sweet drinks at least 5 hours before the scan. The PET scans were performed at 60 min after the intravenous injection of about 200 MBq of FDG. The patients were scanned from the middle of the thigh to the top of the skull.

The images were reconstructed using the ordered subset expectation maximization algorithm and the following parameters: 200x200 matrix, field of view: 815 mm, two iterations, 24 subsets, and a 6-mm Gaussian filter.

### Histopathological parameters

The histopathological parameters of the patients who underwent surgical treatment were determined by examining the surgical specimens. Each type of infiltration (lymphatic, venous, and neural infiltration) was classified using a 4-grade system [18] (0 = no infiltration, 3 = severe infiltration).

### Image analysis

**Fig 1:**
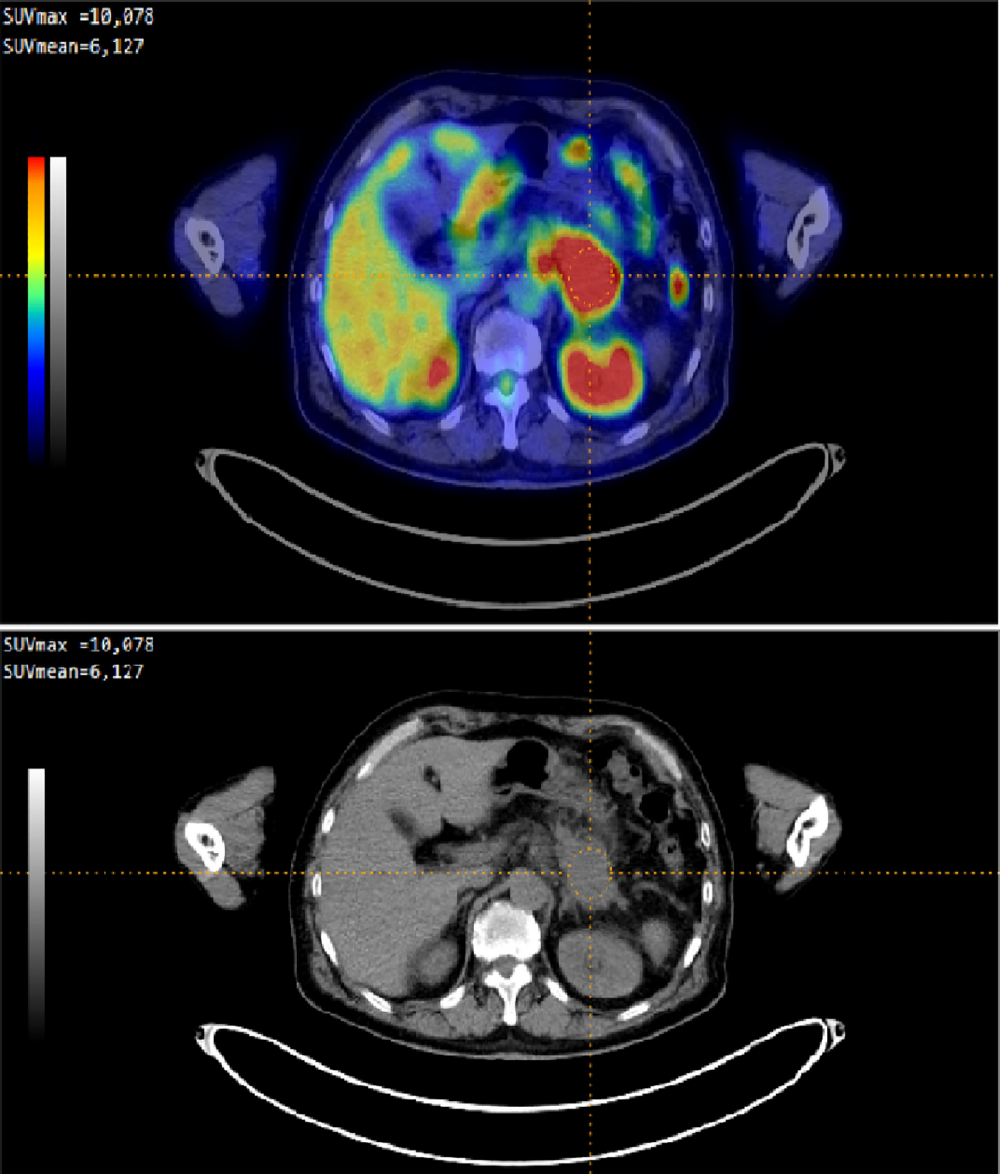
FDG PET imaging results in patient with pancreatic adenocarcinoma using Metavol software. On the right panel, MIP (maximum intensity projection) image of FDG is presented. Cross mark indicates location of tumor. On the left upper panel showing fusion image of FDG and CT (same location of crossmark of right panel). Colors illustrates level of FDG activity. Red area indicates high FDG activity. Pancreatic cancer showing strong uptake. Another high uptake areas are normal kidney and intestine. On the left lower panel showing image of CT (same location of crossmark of right panel).

All FDG PET images were extracted from the electronic archival system at Nagasaki University Hospital and were visually inspected. In each case, after visual qualitative identification of the primary pancreatic lesion, a semi-quantitative evaluation was performed. The SUVmax, SUVpeak, and the MTV of the tumor and the SUVmean of the liver were evaluated on PET images using the Metavol software (Fig 1) [19].

To determine the SUVmax, a spherical volume of interest (VOI), which included the entire lesion in the axial, sagittal, and coronal planes, was examined. CT images were used to ensure that the ^18^F-FDG uptake of normal organs, such as the bowel and stomach, was not included in the VOI. The following PET-related parameters were measured: the SUVmax of the tumor, which was defined as the SUV for the point that exhibited the greatest tracer uptake (the hottest voxel); the SUVpeak of the tumor, which was defined as the average SUV within a 1-cm^3^ spherical volume around the SUVmax; the SUVmean of the liver (as a normal organ); the MTV, which was defined as the volume of the tumor that exhibited FDG uptake; and the TBR, which was defined as the ratio of the tumor’s SUVmax to the SUVmean of the healthy liver (background). To determine the tumor volume, it was necessary to delineate the tumor. To do this, images of the tumor were segmented using the fixed-threshold method, and an SUVmax threshold value of ≥2.5, which was recommended in many previous studies due to its simplicity and objectivity, was used to identify the tumor [20–23]. TLG was calculated as follows: the SUVmean of the tumor x the MTV.

### Statistical analysis

All statistical analyses were performed using the JMP Pro11 software. SUVmax, SUVpeak, the TBR, TLG, and the MTV were compared between each pair of groups using the non-parametric Wilcoxon test. All FDG parameters are expressed as median (1st quartile, 3rd quartile) values. P-values of <0.05 were considered to be statistically significant.

## Results

### Subjects’ characteristics

The subjects’ characteristics are listed in Table 1.

**Table 1:** Total group characteristic.

Parameters	Total (*n = 48*)
Age (mean, range)	68 (40–86)
Gender (M:F)	27:21
SUVmax (median, range)	6.96 (2.526–22.756)
SUVpeak (median, range)	6.03 (2.205–18.903)
TBR (median, range)	3 (1.32–8.221)
MTV (cm^3^) (median, range)	27.04 (0.547–94.75)
TLG (g) (median, range)	114.2 (1.487–504.631)

There were 48 patients (27 males and 21 females), and their mean age was 68 years. Twenty-four patients were considered to have operable disease and underwent surgery. The remaining 24 patients were diagnosed with non-operable disease.

The distribution of TNM staging is presented in Table 2.

**Table 2:** TNM staging of total group.

Tumor staging	Operable group	Non-operable group
**T_0_**	1	0
**T_1_**	2	0
**T_2_**	2	2
**T_3_**	9	4
**T_4_**	10	15
**Lymph node staging**		
**N_0_**	9	8
**N_1_**	7	7
**N_2_**	3	2
**N_3_**	5	2
**M staging**		
**M_0_**	24	16
**M_1_**	0	7

### Characteristics of the operable group

Pancreatic tumors in operable group of patients were located in head– 14 patients (58,4%), body– 5 patients (20,8%) and tail– 5 patients (20,8%).

The resection margin was R1 in 2 patients (8.3%) and R0 in 22 patients (91.7%). None of the patients had macroscopically positive margins.

The distribution of each type of infiltration is presented in Table 3.

**Table 3:** Distribution of types of infiltration.

	Grade 0 (low infiltration)	Grade 1 (mild infiltration)	Grade 2 (moderate infiltration)	Grade 3 (high infiltration)
**Lymphatic**	3	11	8	2
**Venous**	2	6	14	2
**Neural**	4	5	11	4

As there were only small numbers of patients with grade 0 or 3 infiltration, we separated the patients with operable disease into two histological invasiveness groups. The patients with grade 0 or 1 disease were included in “Low invasion” group, and those with grade 2 or 3 disease were included in “High invasion” group. The results of the statistical analyses of the differences in the FDG PET/CT-derived and histopathological parameters between groups are listed in Table 4.

**Table 4:** Difference between “Low invasion” and “High invasion” subgroups in FDG PET parameters and histopathological parameters.

*FDG PET parameters*	Histopathological parameters (infiltration)					
	lymphatic		venous		neural	
	Low invasion group	High invasion group	Low invasion group	High invasion group	Low invasion group	High invasion group
***SUV max***	6.59 (4.17;7.65)	5.94 (4.01;8.49)	4.60 (4.11;5.51)	7.24 (4.63;7.78)	4.61 (3.56;7.77)	6.84 (4.20;7.61)
	P—0.6166		P—**0.0356**		P—0.1195	
***SUV peak***	5.5 (3.54;6.59)	5.22 (3.50;7.01)	3.80 (3.99;6.79)	5.75 (3.99;6.79)	3.99 (3.09;6.67)	5.63 (3.61;6.60)
	P—0.8836		P—**0.0346**		P—0.2572	
***TBR***	2.53 (1.85;3.29)	2.96 (1.87;3.72)	1.92 (1.84;2.04)	3.18 (2.30;3.71)	1.97 (1.50;4.32)	3.09 (2.06;3.69)
	P—0.3564		P—**0.0406**		P—0.0693	
***TLG***	31.60 (14.36;115.03)	48.98 (27.26;125.57)	23.41 (13.98;101.41)	57.94 (27.45;117.37)	24.56 (7.60;136.92)	38.61 (27.33;115.42)
	P—0.4642		P—0.1046		P—0.3107	
***MTV***	8.48 (4.64;30.32)	14.97 (8.69;37.21)	7.66 (4.46;29.02)	15.47 (8.32;31.04)	7.86 (2.61;31.03)	10.15 (8.16;31.40)
	P—0.3340		P—0.1683		P—0.2572	

**Fig 2:**
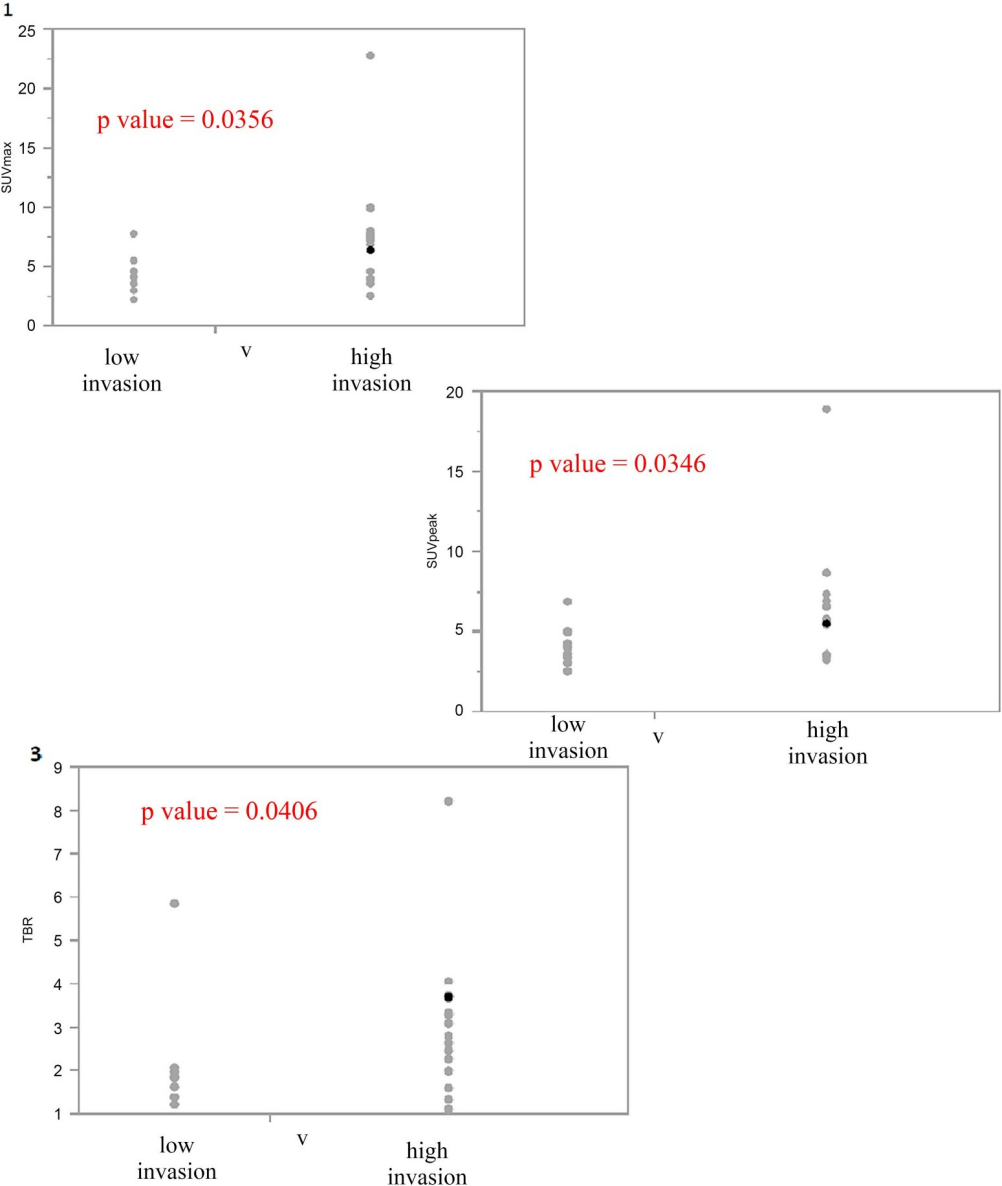
Difference of FDG PET parameters and histopathological parameters in “Low invasion” and “High invasion” subgroups of operable group of patients. 1. Difference of SUVmax and venous infiltration in “Low invasion” and “High invasion” subgroup of patients (p value– 0.0356) 2. Difference of SUVpeak and venous infiltration in “Low invasion” and “High invasion” subgroup of patients (p value– 0.0346). 3. Difference of TBR and venous infiltration in “Low invasion” and “High invasion” subgroup of patients (p value– 0.0406).

In the analysis of venous infiltration, significant differences in the metabolic parameters SUVmax, SUVpeak, and the TBR were detected between groups “Low invasion” and “High invasion” (Fig 2).

SUVpeak exhibited the most significant difference between groups “Low invasion” and “High invasion” (p = 0.0346). On the contrary, neither of the volumetric parameters (TLG and MTV) exhibited significant intergroup differences. None of the FDG PET/CT-derived parameters demonstrated significant intergroup differences in the analyses of lymphatic or neural infiltration.

### Characteristics of the non-operable group

Among 24 patients in non-operable group, main decision-making reasons for non-operability of patients were as follows: locally advanced tumor– 17 patients (70.8%), patient’s decision– 2 patients (8.3%), common bile duct obstruction– 2 patients (8.3%), multiple factors (age, poor general condition, distant meta)– 2 patients (8.3%) and distant metastasis– 1 patient (4.2%).

### Comparison between the operable and non-operable groups

As the operable and non-operable groups contained equal number of patients, the FDG PET/CT-derived parameters of each group were compared.

The results of the statistical analyses of the differences in the FDG PET/CT-derived parameters between the operable and non-operable groups are listed in Table 5.

**Table 5:** Difference between operable and non-operable subgroups.

Parameters	Operable group	Non-operable group	p-value
Gender (M:F)	18:6	9:15	**0.009**
Age, M ±SD	66 ±10	69 ±9	0.268
SUV max	6.4; (4.1; 7.7)	7.7 (4.6; 9.9)	0.083
SUV peak	5.4 (3.6; 6.6)	6.3 (3.9; 8.4)	0.108
TBR	2.5 (1.9; 3.7)	3.1 (2.5; 4.1)	0.155
TLG	35.2 (23.4; 114.6)	108.0 (49.0; 256.8)	**0.035**
MTV	9.8 (7.4; 30.7)	27.0 (14.3; 58.9)	**0.035**

Only the volumetric parameters; i.e., TLG and the MTV, differed significantly between the operable and non-operable groups.

**Fig 3:**
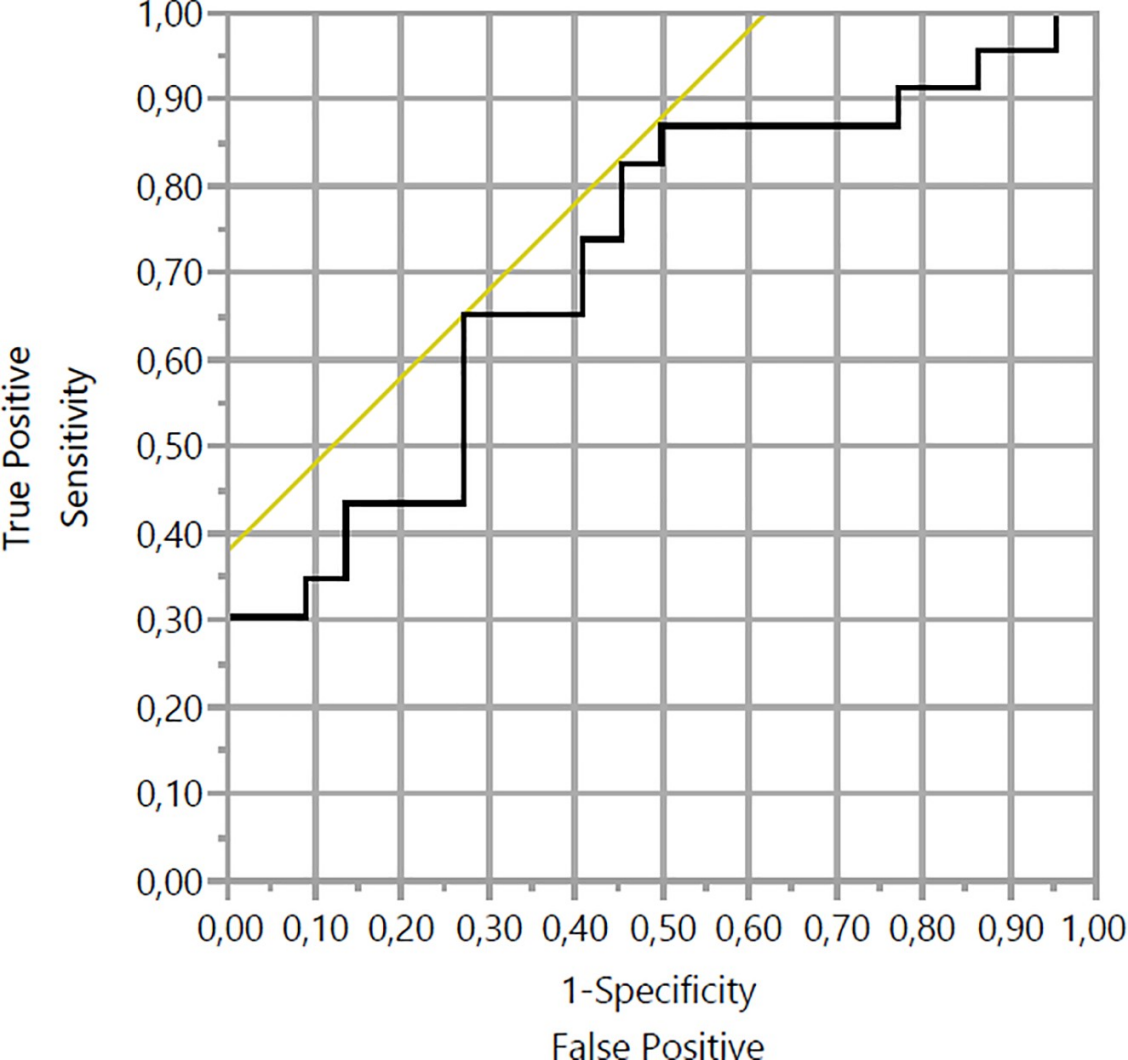
Receiver operating characteristic (ROC) analysis for TLG of pancreatic cancer patients.

**Fig 4:**
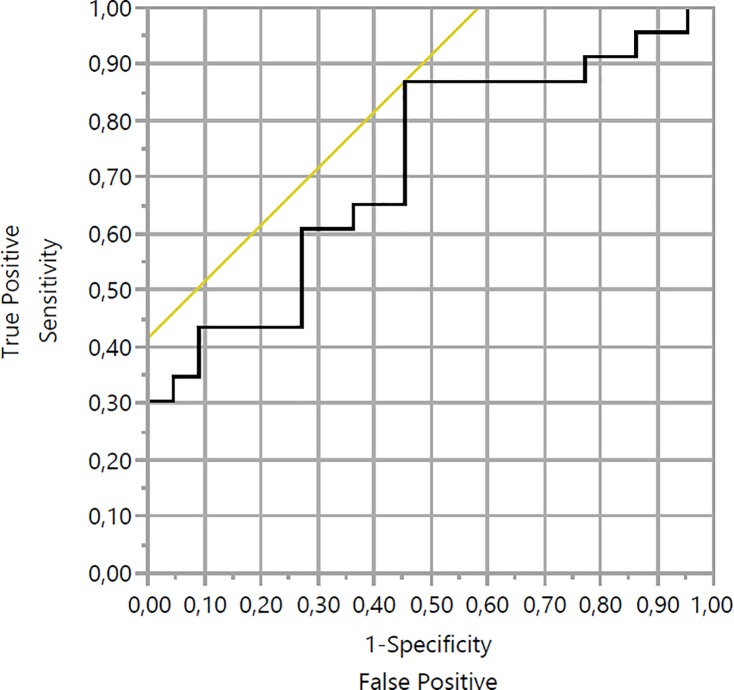
Receiver operating characteristic (ROC) analysis for MTV of pancreatic cancer patients.

Receiver operating characteristic (ROC) analysis was used to calculate the cut off for the decision of non-operability. The ROC curve was drawn for TLG and MTV and the area under the curve was 0.715 (Fig 3, Fig 4).

Using ROC analysis, a cut-off level of TLG was taken at 94,935 g where optimal sensitivity and specificity were 65.2% and 27.3% respectively. For the MTV, ROC analysis showed a cut-off level at 10.6 cm^3^ where optimal sensitivity and specificity were 86.9% and 45.5% respectively.

The results of statistical analysis of correlation between histopathological parameters and clinical outcome are listed in additional materials to article.

## Discussion

In the present study, we found that in pancreatic cancer some metabolic FDG PET/CT-derived parameters differed according to the type/extent of histological invasion detected in the surgical specimens. We also found that the MTV and TLG were associated with operability. Interestingly, there were clear differences between the kinds of FDG PET/CT-derived parameter linked to invasiveness and operability.

There are many FDG PET/CT-derived parameters, and clinicians can become confused about which parameters they should use. Metabolic FDG PET/CT-derived parameters, such as SUV-based parameters and volumetric parameters, such as the MTV and TLG, have been suggested to be prognostic factors for various tumors, including pancreatic cancer [15–17]. The purpose of this study was to evaluate the differences between operable and non-operable cases of pancreatic cancer using FDG PET/CT-derived parameters, and to investigate whether volumetric parameters (TLG and MTV) are superior to SUV-based parameters for assessing the infiltration status/operability of pancreatic cancer.

FDG PET/CT depicts tissue glucose metabolism. FDG uptake is correlated with tumor activity, tumor growth, etc. In other words, FDG uptake is correlated with tumor aggressiveness. This probably explains why FDG PET/CT-derived parameters are correlated with venous infiltration, which is an index of tumor aggressiveness. Several studies have found that vascular infiltration was associated with poor outcomes in patients with resected pancreatic cancer [24], [25].

We identified some parameters that tended to be associated with neural infiltration. For example, in the comparison between groups “Low invasion” and “High invasion” the TBR showed a p-value of 0.06, which is very close to the threshold of significance. The number of cases was probably too small to allow this relationship to reach significance. Several studies have examined the impact of neural infiltration on survival in resected pancreatic adenocarcinoma and reported that such infiltration was a significant prognostic factor. In fact, neural infiltration was found to be associated with poor survival following pancreaticoduodenectomy [24], [26–28].

A systematic review performed by Garcea et al. showed that infiltration into the perineural and vascular tissue reflects an aggressive cancer phenotype. Neural infiltration, in particular, is regarded to be associated with local recurrence in pancreatic cancer and is linked with increasing de-differentiation of pancreatic tumors. Neural infiltration is more common and might be an early event in pancreatic cancer, whereas venous infiltration leads to hematogenous dissemination, and hence, has a greater impact on survival. This difference between neural and venous infiltration might be another reason why we found that FDG PET/CT-derived parameters were useful for predicting venous infiltration, but not neural infiltration [29].

However, none of the examined parameters even exhibited a tendency towards an association with lymphatic infiltration in the present study. The characteristics of venous and neural infiltration are probably different from those of lymphatic infiltration. It is well known that pancreatic cancer is more aggressive than other types of gastrointestinal tract cancer, and it displays a propensity for early dissemination to regional (e.g., the lymph nodes) and distant (e.g., the liver, peritoneum, and lungs) sites. Tumor recurrence after radical surgery is observed in most cases. Such relapse is explained by the early course of lymphogenous metastasis, in which the spread of cancer cells is promoted through the interstitium.

Previous studies [30–33] have indicated that the MTV is a valuable predictor of survival in patients with pancreatic adenocarcinoma. For example, it was reported that the MTV is more strongly correlated with survival than SUVmax. The MTV is defined as the volume of tumor tissue that exhibits FDG uptake above a set threshold, which was set at an SUV of 2.5 in the current study. Previous studies have also demonstrated that TLG is a more accurate predictor of survival than SUVmax or SUVpeak. TLG is indicative of the metabolic activity taking place throughout a cancer lesion, and a large TLG might reflect a small volume of tissue with high FDG uptake or a large volume of tissue with lower FDG uptake.

We found that the MTV and TLG were not related to histopathological parameters. However, given the size of our study, we cannot comment on the true significance of this finding. In addition, we did not detect any correlations between metabolic parameters (SUVmax, SUVpeak, or the TBR) and lymphatic or neural infiltration.

On the other hand, when we compared the operable and non-operable subgroups, we found intergroup differences in the MTV and TLG. These results indicated that the patients that displayed higher values for volumetric parameters tended to have non-operable disease, but among the surgically treated patients those that displayed higher values for volumetric parameters did not tend to exhibit greater infiltration. Instead, only high-activity tumors tended to display such invasive behavior. We consider that this discrepancy indicates that it is important to carefully assess tumor aggressiveness. Patients with large TLG values, but low SUV, might only have limited infiltration, and so their disease might be operable. On the other hand, patients with small TLG values, but high SUVmax, might have inoperable disease due to hidden infiltration.

Receiver operating characteristic analysis showed, that for the patients whose values of MTV and TLG higher than threshold, decision of non-operability can be reasonable, especially for the MTV which showed relatively high sensitivity. However, considering low specificity, we can’t consider, that lower than threshold is decision for performing surgery. Decision for performing of surgery is depend not only from tumor condition, but also from general condition of patient, concomitant diseases, patient’s decision etc. So, we consider, that MTV and TLG can be helpful for taking decision to perform surgery, but not as main reason.

We recognize that our study has several limitations. First, the FDG PET/CT images and patient follow-up data were analyzed retrospectively. In addition, we did not analyze the patients’ prognoses. Second, decision of operability was retrospective, taken by clinical doctor and strongly influenced by clinical information such as age, general condition etc. We noticed that many of our subjects are borderline operability and it is hard to decide operability completely free from clinical information. Thus, we did not perform such re-classification. Third, the number of patients with pancreatic adenocarcinoma was small, which might have limited our conclusions. Fourth, the VOI were drawn and analyzed by two radiologists based on a consensus approach. We did not assess the inter- or intraobserver variability in the placement of the VOI or its effects on our results.

## Conclusion

In conclusion, metabolic ^18^F-FDG PET/CT-derived parameters, such as SUVmax, SUVpeak, and the TBR, are useful for predicting venous infiltration status in patients with operable pancreatic adenocarcinoma. Nevertheless, volumetric FDG PET/CT parameters, such as the MTV and TLG, can be useful for reaching clinical decisions regarding the operability of pancreatic tumors.

## Supporting information

S1 Fig Kaplan-Meier analysis for Lymphatic infiltration and clinical outcome. (PDF)

S2 Fig Kaplan-Meier analysis for Neural infiltration and clinical outcome. (PDF)

S3 Fig Kaplan-Meier analysis for Venous infiltration and clinical outcome. (PDF)

S1 Data Statistical data. (XLSX)
