# Supplementary figures and images for: Usefulness of FDG PET/CT derived parameters in prediction of histopathological finding during the surgery in patients with pancreatic adenocarcinoma
Source: PLoS One. 2019 Jan 10;14(1):e0210178. doi: 10.1371/journal.pone.0210178 (PMC6328180; doi:10.1371/journal.pone.0210178)

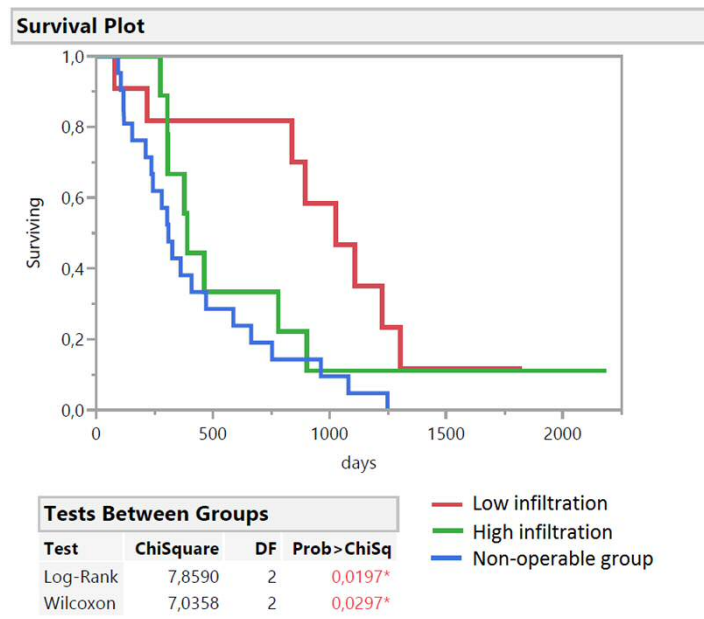

Fig S1. Kaplan-Meier analysis for Lymphatic infiltration and clinical outcome.

Supplement: S1 Fig — (PDF) [file pone.0210178.s001.pdf]

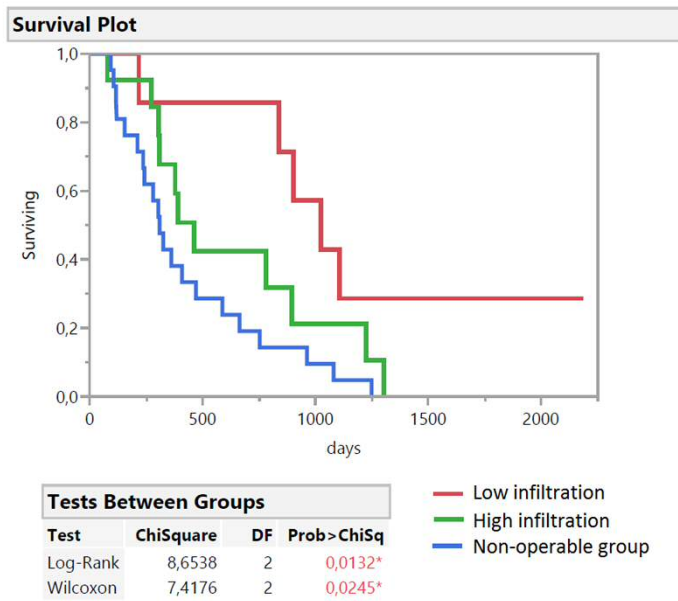

Fig S2. Kaplan-Meier analysis for Neural infiltration and clinical outcome.

Supplement: S2 Fig — (PDF) [file pone.0210178.s002.pdf]
