# Supplementary material for: Usefulness of FDG PET/CT derived parameters in prediction of histopathological finding during the surgery in patients with pancreatic adenocarcinoma
Source: PLoS One. 2019 Jan 10;14(1):e0210178. doi: 10.1371/journal.pone.0210178 (PMC6328180; doi:10.1371/journal.pone.0210178)
Supplement: S3 Fig — (PDF) [file pone.0210178.s003.pdf]

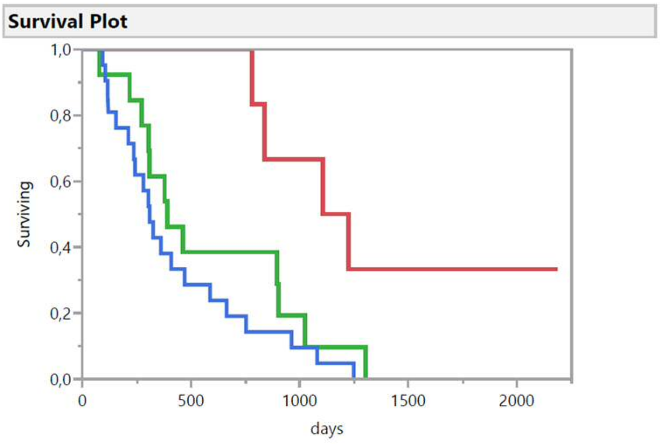

Tests Between Groups

Test	ChiSquare	DF	Prob>ChiSq
Log-Rank	10,4960	2	0,0053*
Wilcoxon	9,4209	2	0,0090*

Fig S3. Kaplan-Meier analysis for Venous infiltration and clinical outcome.

Kaplan-Meier analysis showed strong correlation between histopathological parameters and clinical outcome. Tests between groups of patients with low infiltration, high infiltration and non-operable group of patients showed significant difference for lymphatic (p value – 0.0297), neural (p value – 0.0245) and venous infiltration (p value – 0.0090).

Several previous studies, have found that higher infiltration have tendency to poor prognosis. Results of our study are support results of previous studies.
